# The development of the adult intestinal stem cells: Insights from studies on thyroid hormone-dependent amphibian metamorphosis

**DOI:** 10.1186/2045-3701-1-30

**Published:** 2011-09-06

**Authors:** Yun-Bo Shi, Takashi Hasebe, Liezhen Fu, Kenta Fujimoto, Atsuko Ishizuya-Oka

**Affiliations:** 1 Section on Molecular Morphogenesis, Laboratory of Gene Regulation and Development, Program in Cellular Regulation and Metabolism (PCRM), Eunice Kennedy Shriver National Institute of Child Health and Human Development (NICHD), National Institutes of Health (NIH), Bethesda, Maryland, 20892, USA; 2 Department of Biology, Nippon Medical School, Kawasaki, Kanagawa 211-0063, Japan

## Abstract

Adult organ-specific stem cells are essential for organ homeostasis and repair in adult vertebrates. The intestine is one of the best-studied organs in this regard. The intestinal epithelium undergoes constant self-renewal throughout adult life across vertebrates through the proliferation and subsequent differentiation of the adult stem cells. This self-renewal system is established late during development, around birth, in mammals when endogenous thyroid hormone (T3) levels are high. Amphibian metamorphosis resembles mammalian postembryonic development around birth and is totally dependent upon the presence of high levels of T3. During this process, the tadpole intestine, predominantly a monolayer of larval epithelial cells, undergoes drastic transformation. The larval epithelial cells undergo apoptosis and concurrently, adult epithelial stem/progenitor cells develop *de novo*, rapidly proliferate, and then differentiate to establish a trough-crest axis of the epithelial fold, resembling the crypt-villus axis in the adult mammalian intestine. We and others have studied the T3-dependent remodeling of the intestine in *Xenopus laevis*. Here we will highlight some of the recent findings on the origin of the adult intestinal stem cells. We will discuss observations suggesting that liganded T3 receptor (TR) regulates cell autonomous formation of adult intestinal progenitor cells and that T3 action in the connective tissue is important for the establishment of the stem cell niche. We will further review evidence suggesting similar T3-dependent formation of adult intestinal stem cells in other vertebrates.

## Introduction

Organ-specific adult stem cells are essential for the development of adult organs and tissue repair and regeneration. While most vertebrates develop directly into the adult form by birth, their organ development often involves a two-step process, the formation of an immature but often functional organ during embryogenesis followed by the maturation into the adult form. This second step takes place during the so-called post-embryonic development, a period around birth in mammals such as human and mouse when plasma thyroid hormone (T3) concentrations are high [1]. The organ-specific adult stem cells are often formed/matured during this period. One of the well-studied such organs is the intestine. The tissue responsible for the main physiological function of the intestine, the intestinal epithelium, which is responsible for the food processing and nutrient absorption, is continuously renewed throughout adult life in vertebrates. This takes place through stem cell divisions in the crypt, followed by their differentiation as the cells migrate up to and along the villus and eventual death of the differentiated cells near the tip of the villus. In adult mammals, the intestinal epithelium is replaced once every 1-6 days [2-4], and in amphibians, this occurs in 2 weeks [5]. Such a self-renewal system has been shown to be present throughout vertebrates, from zebrafish, frogs, to human. While a number of signaling pathways have been shown to be important for intestinal development and cell renewal in the adult [4,6], much less is known about how adult stem cells are formed during development, in part due to the difficulties to study the uterus-enclosed mammalian embryogenesis.

Intestinal remodeling during amphibian metamorphosis offers a unique opportunity to study the development of adult organ-specific stem cells in vertebrates. As during postembryonic development in mammals, T3 levels in the plasma are high during amphibian metamorphosis. In fact, T3 is both necessary and sufficient for premetamorphic tadpoles to transform into frogs [7,8]. In premetamorphic tadpoles, there is little T3. The synthesis of endogenous T3 around stage 55 in *Xenopus laevis *initiates metamorphosis. The plasma T3 rises to peak levels at the climax of metamorphosis and subsequently is reduced to much lower levels by the end of metamorphosis. During metamorphosis, different organs undergo vastly different changes, including total resorption such as the tail and gills, *de novo *development such as the limb, and drastic remodeling such as the liver, pancreas and intestine, which involve both larval cell death and adult cell development. Despite such complex changes, all these changes are controlled by T3. An important advantage of this system is that it occurs independent of maternal influence as in the case of mammals. Furthermore, this process can be induced even in organ cultures of premetamorphic tadpoles when treated with physiological concentrations of T3 [7,8]. This makes it easy to manipulate and study the development and regulation of the adult organ-specific stem cells.

**Figure 1:**
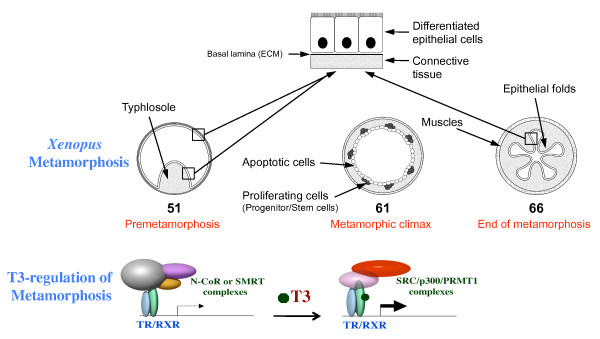
**T3-dependent intestinal remodeling during *Xenopus laevis *metamorphosis serves as a model to study adult organ-specific stem cell development in vertebrates**. Top: In premetamorphic tadpoles, there is little or no T3 and the intestine has a simple structure with only a single fold, the typhlosole. This structure is similar to the mammalian embryonic intestine. At the metamorphic climax when T3 level is high, the larval epithelial cells begin to undergo apoptosis, as indicated by the circles. Concurrently, the proliferating adult progenitor/stem cells develop *de novo *from larval epithelial cells through dedifferentiation, as indicated by black dots. By the end of metamorphosis, the newly differentiated adult epithelial cells form a multiply folded epithelium, similar to mammalian adult intestines. This process is entirely controlled by T3 and can be induced even in organ cultures of tadpole intestine with T3 treatment. In mammals, the intestine undergoes postembryonic maturation into the adult form around or shortly after birth when T3 levels are also high. Thus, intestinal metamorphosis offers a unique opportunity to study the development of adult intestinal stem cells. Bottom: T3 functions by regulating gene transcription through TRs. In the absence of T3 (as in premetamorphic tadpole), TR forms heterodimers with RXR and the heterodimer binds to target gene promoters to repress their expression by recruiting corepressor complexes containing the related proteins N-CoR or SMRT and histone deacetylases. When T3 is present, the corepressor complexes are released upon T3 binding to TR, and simultaneously coactivator complexes such as those containing SRC, p300, and PRMT1, are recruited to activate target gene expression. SRC and p300 are histone acetyltransferases and PRMT1 is a histone methyltransferase. The coactivator complexes will modify histones and activate gene expression to induce metamorphosis.

In the South African clawed toad *Xenopus laevis*, the tadpole intestine consists of largely a monolayer of larval epithelial cells, with thin layers of surrounding connective tissue and muscles [9] (Figure 1). When T3 becomes available either endogenously or exogenously, the vast majority, if not all, of the larval epithelial cells undergo apoptosis and concurrently, adult epithelial stem/progenitor cells appear *de novo *and rapidly proliferate [9-11]*(Note that organ-specific stem cells are traditionally defined for adult organs. It is possible that the developing stem cells during organogenesis are not the same as in adult organs. For simplicity, we will refer the proliferating adult epithelial cells with detectable stem cell markers during metamorphosis to as stem cells while those lacking the stem cell markers as progenitors)*. These adult epithelial cells subsequently differentiate to establish a trough-crest axis of epithelial folds by the end of metamorphosis, resembling the crypt-villus axis in the adult mammalian intestine, accompanied by the maturation of the connective tissue and muscles [9]. Just like all other processes during metamorphosis, all these changes during intestinal remodeling are controlled by T3 and can be induced even in organ cultures treated with T3 [10,12,13], indicating organ-autonomous formation of adult stem/progenitor cells. Such interesting properties have allowed studies to determine the origin of the stem cells and investigate the mechanisms governing the formation of the adult stem cells.

### Mechanism of the regulation of *Xenopus *development by T3

T3 binds to T3 receptors (TRs) with high affinities. TRs are transcription factors that form heterodimers with 9-cis retinoic acid receptors (RXRs) and these dimers bind to T3 response element (TRE) in/around the promoters of T3 target genes [14-17]. For T3-inducible genes, TR/RXR functions as a repressor in the absence of T3 and as an activator in the presence of T3. TRs recruit different cofactor complexes to TREs to affect transcription [15,18-25]. Extensive molecular and genetic studies by a number of different laboratories have shown that TR is both necessary and sufficient to mediate the metamorphic effects of T3 and that TR has dual functions during *Xenopus *development [26-35]. In premetamorphic *Xenopus laevis *tadpoles, TR recruits corepressor complexes to target genes when T3 is absent and this recruitment is important to keep the T3-inducible genes repressed, thus regulating metamorphic timing (Figure 1) [31,36-38]. After stage 55, when endogenous T3 becomes available, corepressor complexes are released and coactivator complexes are recruited by TR, this leads to the activation of T3 target genes and metamorphosis [33,34,39-43].

### The origin of the adult intestinal epithelial stem cells

Earlier microscopic and cytological studies of intestinal remodeling in *Xenopus laevis *failed to identify any adult stem cells in premetamorphic tadpoles [9]. On the other hand, the ability to induce intestinal remodeling in intestinal organ cultures [10,12,13] indicates that the adult stem cells develop *de novo *within tadpole intestine. It is possible that non-epithelial cells may give rise to the stem cells when T3 becomes available. Alternatively, some differentiated larval epithelial cells may undergo dedifferentiation to become the stem cells during metamorphosis. This latter scenario is supported by chronological observations of intestinal metamorphosis [44] and the apparent presence of the proteins of the differentiated epithelial cells in proliferating adult progenitor cells [11,45]. On the other hand, there has been no direct evidence to demonstrate the origin of the adult epithelial cells. Making use of a transgenic *Xenopus *line ubiquitously expressing the green fluorescent protein (GFP), we have recently carried out recombinant organ culture studies to determine the orgin of the adult intestinal epithelial stem cells [10].

**Figure 2:**
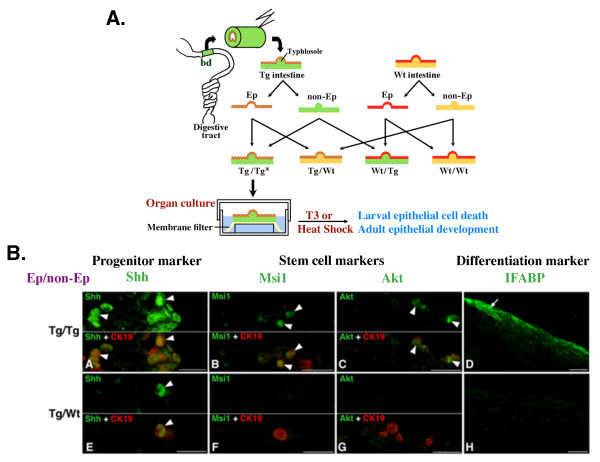
**Recombinant intestinal organ culture studies using transgenic tadpole**. **A. Schematic diagram for tissue recombination and organ culture of the *Xenopus laevis *intestine**. Transgenic (Tg) frogs are generated using a double promoter construct [61] where one GFP is expressed in the lens of the eye under the control of the γ-crystallin gene promoter for identification of the transgenic animals and another GFP is expressed ubiquitously under the CMV promoter or a dominant positive thyroid hormone receptor (dpTR, which resembles T3-bound TR, or activated TR, but does not need T3) is expressed under the control of a heat shock inducible promoter. The adult Tg frogs are used to produce Tg and wild type (Wt) sibling tadpoles. Tubular fragments are isolated from the small intestine just behind the bile duct junction (bd) of premetamorphic (when there is little endogenous T3 present) Tg and Wt tadpoles, slit open lengthwise, and separated into epithelium (Ep) and non-epithelial tissues (non-Ep), which consists of mainly the connective tissue underlying the epithelium. Each Ep is then recombined with homologous or heterologous non-Ep. The four kinds of recombinant intestines are placed on membrane filters on grids and cultured *in vitro*. Heat shock treatment, when using dpTR tadpoles, or T3 treatment, when using GFP animals, is applied to induce intestinal remodeling. * indicated type of Ep/type of non-Ep used in the recombinant organ culture: e.g., Tg/Wt indicating Tg epithelium recombined with Wt non-epithelial tissues. **B. Recombinant organ culture studies using dpTR-transgenic and Wt tadpoles indicate that only when both the Ep and non-Ep were derived from Tg animals, i.e., Tg/Tg, were true stem cells and adult epithelium formed after heat shock treatment**. Recombinants made of Tg EP and Tg non-EP (Tg/Tg) (A-D) and Tg Ep and Wt non-EP (Tg/Wt) (E-H) of the intestines were cultured with heat shock treatment for 5 (A-C, E-G) or 7 (D, H) days *in vitro*. Cross sections were double-immuno-stained with anti-Shh (green, sonic hedgehog, an adult progenitor cell marker) and anti-CK19 (red, cytokeratin-19, which is expressed in epithelial cells. (A, E)), anti-Msi-1 (green, Musashi-1, a stem cell marker of the vertebrate adult intestine) and anti-CK19 (red; (B, F)), or anti-Akt (green, a stem cell marker of the vertebrate adult intestine) and anti-CK19 (red) antibodies (C, G), or immunostained with anti-IFABP antibody (green; D, H). In both Tg/Tg (arrowheads; (A)) and Tg/Wt intestines (E), cells positive for Shh become detectable on day 5 among cells expressing CK19, indicating the adult progenitor cells can be induced by cell-autonomous action of activated TR in the epithelium. Cells positive for Msi1 and Akt are also detected among CK19- immunoreactive cells in Tg/Tg intestine (arrowheads; (B, C)) but not in Tg/Wt intestine (F, G). In addition, differentiated epithelial cells expressing IFABP were present after extended culturing only in Tg/Tg organ cultures (D). Thus, activation of TR in the non-epithelial tissues is also required for the stem cell formation, likely by contributing to the formation of the stem cell niche. Not shown here is that most of the epithelial cells undergo apoptosis when dpTR is expressed in either the EP or non-EP or both, just like that during metamorphosis when T3 binds to TR. See [47] for details.

As diagramed in Figure 2A, a fragment of the small intestine near the bile duct was isolated from GFP-expressing transgenic as well as wild type premetamorphic tadpoles. The epithelium and non-epithelial tissues, which are made of mainly the fibroblasts in the connective tissue, can be enzymatically separated from each other. The transgenic and wild type tissues were recombined into 4 types of recombinant intestinal organ cultures and treated with a physiological concentration of T3, which induced intestinal remodeling just like in intact animals, namely, larval epithelial cell death and adult epithelium development. In all recombinant intestines, adult progenitor cells expressing markers for intestinal progenitor/stem cells such as sonic hedgehog (Shh) became detectable and then differentiated into the adult epithelium expressing intestinal fatty acid binding-protein (IFABP), a marker for absorptive cells. Importantly, whenever the epithelium was derived from transgenic intestine, both the adult progenitor/stem cells and differentiated cells expressed GFP, while neither of them expressed GFP if the epithelium was derived from wild type tadpoles [10]. These results thus demonstrate that the intestinal progenitor/stem cells that give rise to the adult intestinal epithelium originate from the larval epithelium, likely through T3-induced dedifferentiation of some larval epithelial cells.

### Distinct roles of the epithelium and connective tissue in stem cell formation

Earlier organ culture studies have shown that adult epithelium formation requires the presence of the connective tissue [46]. How the connective tissue participates in this process remains largely unknown. Given that the adult epithelial stem cells originate from the larval epithelium, it is possible that T3 induces the formation of the stem cells in a cell-autonomous manner. To investigate whether T3 signaling in the epithelium alone is sufficient for adult stem cell formation, we have carried out recombinant organ culture experiments by using premetamorphic transgenic *Xenopus laevis *tadpoles that express a dominant positive TR (dpTR, which is constitutively active but does not bind to T3) under the control of a heat shock inducible promoter [29,47] (Figure 2A). Heat shock treatment of the recombinant intestinal organ cultures leads to the expression of dpTR only in the transgenic but not wild type tissues while the endogenous TR remains unliganded regardless of the treatment. When either the epithelium or the non-epithelial tissues or both were derived from the transgenic tadpoles, larval epithelial cell apoptosis could be detected after heat shock treatment for 5 days. On the other hand but expectedly, no metamorphic changes were observed after the heat shock treatment when both the epithelium and non-epithelial tissues were from the wild type animals [47]. Thus, larval epithelial cell death can occur via two pathways: suicide cell-autonomously through T3 action in the epithelial cells versus murder through T3 action in the non-epithelial tissues, which likely involves the induction of matrix metalloproteinases such as stromelysin-3, an MMP secreted by fibroblasts that causes epithelial cell death [48].

Adult epithelial progenitor cells expressing Shh were also detectable after 5 days of heat shcok treatment if the recombinant was made of epithelium derived from transgenic intestine, regardless of the origin of the non-epithelial tissues [47]. However, no such cells were present if both tissues were derived from the wild type animals or when the epithelium was from the wild type animals and the non-epithelial tissues were from the transgenic animals. Thus, TR activation in the non-epithelial tissues is not sufficient to induce the formation of the Shh+ adult progenitor cells in the epithelium, although it can induce epithelial apoptosis. The formation of the Shh+ progenitor cells appears to be cell-autonomous upon activation of TR (due to T3 binding or the expression of a constitutively active TR) in the epithelium [47].

When the expression of two stem cell markers of the adult mammalian intestine, Musashi-1 (Msi-1) and Akt [49-51], were analyzed after 5 days of heat shock treatment, they were detected in the Shh+ cells when both of the epithelium and non-epithelial tissues used to make the recombinant intestine were derived from dpTR-transgenic tadpole intestine (Figure 2B). Upon prolonged culturing (5 days of heat shock treatment followed by 2 days of culture without heat shock treatment), such organ cultures formed adult epithelium expressing IFABP, a marker for the absorptive epithelium (Figure 2B). On the other hand, if only the epithelium was derived from the transgenic tadpoles, the Shh+ progenitor cells produced after heat shock treatment failed to express either of the stem cell markers and failed to develop into differentiated adult epithelium expressing IFABP (Figure 2B). These results indicate that while the adult progenitor/stem cells can be induced by T3 in the larval epithelium, liganded TR-mediated gene expression in the surrounding tissues other than the epithelium is required for them to develop into adult stem cells, suggesting that T3 activation of the non-epithelial tissues, most likely the underlying connective tissue is required for the establishment of the stem cell niche in the amphibian intestine during metamorphosis. Consistently, an earlier transgenic study where a dominant negative TR was expressed under the control of epithelial-, fibroblast-, and muscle-specific gene promoters showed that while mutant TR expression in different tissues caused distinct phenotypes in the postmetamorphic intestine, its expression in either the epithelium and fibroblasts led to abnormal epithelia and mesenchyme development [35], supporting our conclusion that TR activation in both the epithelium and connective tissue is important for adult epithelium development.

### A role of the TR-coactivator protein arginine methyltransferase 1 (PRMT1) in stem cell formation during metamorphosis by T3

**Figure 3:**
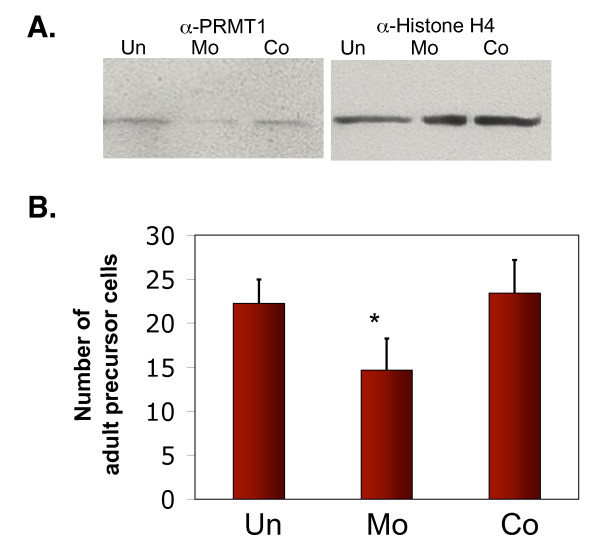
**PRMT1 is required for the development and/or proliferation of adult intestinal progenitor/stem cells**. **A. *In vivo *knock-down of PRMT1 expression**. *Xenopus laevis *tadpoles at stage 53/54 were injected with (MO, Co) or without (Un) PRMT1 (MO) or control (Co) anti-sense Vivo-morpholino oligos for 4 consecutive days. Intestines were dissected for western blot analysis of the expression of the endogenous PRMT1 or histone H4 (as a control). **B. Knocking-down the expression of endogenous PRMT1 results in the reduction in the number of proliferating adult cells in the intestine**. Stage 53/54 premetamorphic tadpoles were injected without (untreated) or with PRMT1 (MO) or control Vivo-morpholino oligos for four days. All tadpoles were then treated with 5 nM of thyroid hormone for 4 days. The tadpoles were sacrificed and the intestine was analyzed for the number of proliferating adult cells. See [53] for details.

As described above, TR functions by recruiting coactivator complexes in the presence of T3 to induce gene regulation and metamorphosis. Earlier studies have shown that the coactivator complexes containing SRC3, p300, and PRMT1, are essential for metamorphosis in *Xenopus laevis *[39-43]. Interestingly, the expression of PRMT1 is upregulated in the intestine during metamorphosis. Kinetic studies of the induction of PRMT1 expression by T3 suggest that it is indirectly regulated by TR. *In situ *hybridization analysis have revealed that it is first upregulated in a small number of larval epithelial cells at early stages of metamorphosis when T3 levels are still low and that these cells appear to dedifferentiate into adult stem cells [52,53]. More importantly, in transgenic tadpoles overexpressing PRMT1 under a heat shock inducible promoter, heat shock treatment led to an increase in the number of progenitor/stem cells in the intestine during natural metamorphosis [53]. On the other hand, knocking down the expression of endogenous PRMT1 with an antisense morpholino oligonucleotide reduced the number of such cells during T3-induced metamorphosis (Figure 3) [53]. Thus, PRMT1 likely plays a role in the formation and/or proliferation of the intestinal progenitor/stem cells, possibly via its coactivator function for liganded TR.

### Evolutionary conservation in adult intestinal stem cell development

**Figure 4:**
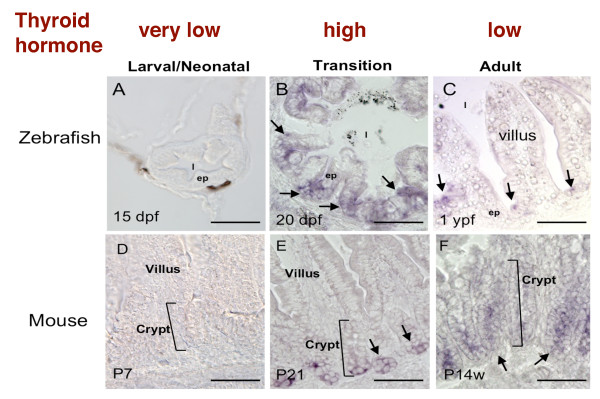
**Conserved spatiotemporal expression patterns of PRMT1 in the postembryonic intestines of fish and mouse suggest a conserved role of PRMT1 in adult intestinal stem cell development**. PRMT1 mRNA was analyzed by *in situ *hybridization in the intestines of zebrafish at 15 days post fertilization (dpf) (A), 20 dpf (B) and 1 year post fertilization (ypf) (C) and mouse at postnatal day 7 (D, P7), P21 (E), and postnatal week 14 (F, P14w). Arrows indicate PRMT1 positive cells in the intestinal epithelium. Note that high levels of PRMT1 expression is detected only in the proliferating/stem cells located in the crypts in both species, resembling that in *Xenopus laevis*. In addition, the transition period in both species when PRMT1 is upregulated is characterized by high levels of T3 in the serum, just like that during *Xenopus *metamorphosis. ep, epithelium. l, lumen. Bars, 50 μm. See [53] for details.

The intestine is physiologically and structurally conserved in adult vertebrates, with the proliferating stem cells located in the crypts/troughs of the epithelial folds and differentiated epithelial cells located along the villi or the folds. At the tip of the villus or fold, the differentiated epithelial cells degenerate through apoptosis. The maturation of this adult structure occurs at different stage of development in different animals but interestingly all occurring around the time when plasma T3 levels are high, just like during amphibian metamorphosis. T3 or TR deficiency leads to abnormal intestinal morphology, a decrease in the number of epithelial cells along the crypt-villus axis and in proliferating crypt cells [54-57]. It has been shown that TRα1 controls intestinal development during maturation at weaning as well as intestinal homeostasis in adulthood through the activation of the proliferation of intestinal progenitors by liganded TRα1 [58]. Thus, T3 and TR appear to have conserved roles in regulating the formation of the adult intestine in mouse and frog. Interestingly, PRMT1 has a conserved spatiotemporal expression pattern during postembryonic intestinal development in vertebrates. During mosue and zebrafish development, little PRMT1 expression was detected in the larval/neonatal intestine in zebrafish or mouse when plasma T3 levels were low (Figure 4A, D). During the transition to the adult intestine when T3 levels were high [59,60], high levels of PRMT1 mRNA were present but only in the bottom of the developing epithelial fold in zebrafish (Figure 4B) or the crypt of the mouse intestine (Figure 4E). In adult animals, the PRMT1 expression was high only in the mouse crypts and the bottom of the zebrafish intestinal epithelial folds, where the stem cells were located (Figure 4C, F). These results suggest that like during *Xenopus *metamorphosis, T3 likely regulates the development of the adult epithelial progenitor/stem cells in zebrafish and mouse intestine and that PRMT1 plays a role in this process. They also suggest that although neonatal mouse intestine is structurally fully developed, with the proper crypt-villus organization similar to that in the adult mouse, the embryonic/neonatal mouse intestinal stem cells are molecularly distinct from those in the adult mouse intestine. Thus, the formation of the adult intestinal stem cells may utilize conserved mechanisms, such as dependence on T3 and involvement of PRMT1, during vertebrate development.

## Conclusion

Organ-specific adult stem cells play an essential role in organ homeostasis and tissue repair and regeneration. Understanding the mechanisms of their development is undoubtedly important for the generation and utilization of such cells for tissue replacement therapies and prevention of stem cell-related diseases. The uterus-enclosed development of mammalian embryos has hindered the study of the development of adult organ-specific stem cells in mammals. On the other hand, the conservation of the organ function and development makes it possible to use non-mammalian models for such purpose. The intestine is such an organ that is structurally and physiologically conserved across vertebrate species. Using *Xenopus laevis *metamorphosis as a model, we and others have shown that the adult intestinal stem cells develop *de novo *and are distinct molecularly from the larval cells. It is interesting to note that larval epithelial cells in premetamorphic tadpole intestines are mitotically active, even though they are differentiated and serve the physiological function of the intestinal epithelium [5]. Thus, to develop into stem cells, the larval epithelial cells merely need to repress the expression of the genes associated with differentiated cells and escape the T3-induced apoptotic fate that most other epithelial cells have. Intestinal organ culture studies suggest that T3 induces the epithelium to undergo cell-autonomous formation of adult progenitor cells and also induces the non-epithelial tissues, most likely the connective tissue, to form or contribute to the establishment of the adult stem cell niche. It is possible that some of the larval epithelial cells, in the context of the stem cell niche formed with the participation of the T3-activated connective tissue, adopt the pathway of stem cell development during metamorphosis. The vast majority of the larval epithelial cells undergo apoptosis due to the lack of the proper stem cell niche environment even though they also lose the expression of at least some differentiation markers such as IFABP. The studies with PRMT1 have not only revealed an important role of this TR-coactivator in adult stem cell development/proliferation but also provided further evidence that adult stem cell development utilize conserved mechanisms across vertebrates.

## Competing interests

The authors declare that they have no competing interests.

## Authors' contributions

YBS, TH, and AI-O wrote the manuscript, LF and KF participated in the discussion and revision. All authors read and approved the final manuscript.
